# Effects of insulin infusion volume on diabetic ketoacidosis outcome in pediatrics: Retrospective observational study

**DOI:** 10.1097/MD.0000000000042453

**Published:** 2025-05-09

**Authors:** Hessa Al Muqati, Abeer Alsmari, Mohanned Alrahili, Fahad AlJuraibah, Omar Aldibasi, Nada A. Alsaleh

**Affiliations:** a Pharmaceutical Care Services, Ministry of the National Guard – Health Affairs, Riyadh, Saudi Arabia; b College of Pharmacy, King Saud bin Abdulaziz University for Health Sciences, Riyadh, Saudi Arabia; c King Abdullah International Medical Research Center, Riyadh, Saudi Arabia; d Neonatal Intensive Care Department, Women’s Health Hospital, Ministry of National Guard Health affairs, Riyadh, Saudi Arabia; e Pediatric Department, King Abdulaziz Medical City, King Abdullah Specialist Children’s Hospital, Ministry of National Guard Health Affairs, Riyadh, Saudi Arabia; f College of Medicine, King Saud bin Abdulaziz University for Health Sciences, Ministry of National Guard Health Affairs, Riyadh, Saudi Arabia; g King Abdullah International Medical Research Center, Ministry of National Guard Health Affairs, Riyadh, Saudi Arabia; h Department of Biostatistics, King Abdullah International Medical Research Center, Riyadh, Saudi Arabia; i College of Dentistry, King Saud bin Abdulaziz University for Health Sciences, Riyadh, Saudi Arabia; j Department of Pharmacy Practice, College of Pharmacy, Princess Nourah Bint Abdulrahman University, Riyadh, Saudi Arabia.

**Keywords:** diabetic ketoacidosis, fluids, hyperchloremia, insulin, pediatrics

## Abstract

Diabetic ketoacidosis (DKA) poses a significant risk to diabetic pediatric patients, warranting effective management strategies to prevent complications. Current DKA management protocols often use normal saline for insulin infusion, which can contribute to hyperchloremia. This study aimed to compare the effects of 2 insulin concentrations (1 unit of regular human insulin per 1 mL vs 1 unit per 10 mL of 0.9% sodium chloride) on the incidence of hyperchloremic metabolic acidosis (HMA). A retrospective observational study was conducted on pediatric patients admitted to a large pediatric hospital, recognized as a leading provider of tertiary pediatric care in the region, from January 2018 to June 2023. The study compared preprotocol (1 unit/10 mL) and postprotocol (1 unit/1 mL) insulin concentrations. Data collected included demographics, DKA severity, electrolytes, intravenous fluid type, total volume, chloride load, and length of hospital stay. The study included 279 DKA admissions, with 140 preprotocol and 139 postprotocol cases. The postprotocol group had a higher incidence of new onset of type 1 diabetes cases. The incidence of HMA was 38% (53 patients) in the postprotocol group, compared with 43% (60 patients) in the preprotocol group, but this difference was not statistically significant (*P* = .365). Furthermore, the total chloride load per kg in the first 24 hours showed no statistical significance between the pre- and postgroups (mean 11 ± 5 and 11 ± 5, respectively; *P* = .665). Similarly, length of stay also showed no significant difference between the pre- and postprotocol groups (mean 3 ± 2 vs 4 ± 3, respectively; *P* = .102). This study examines the comparative effectiveness of 2 insulin concentrations in DKA treatment. Changing insulin concentrations did not influence the incidence of HMA.

## 1. Introduction

Diabetic ketoacidosis (DKA) is a serious complication of type 1 diabetes and it has a mortality rate of 0.3% to 0.5%.^[1]^ Globally, over 108,200 children are diagnosed with type 1 diabetes mellitus (T1DM) each year,^[2]^ with 13% to 80% of them presenting with DKA at the time of diagnosis.^[3]^ Notably, Saudi Arabia demonstrates one of the highest prevalence rates of DKA at 59%.^[4]^ DKA is caused by a reduction in circulating insulin levels and a rise in counterregulatory hormone levels.^[5]^ These disruptions lead to a series of negative consequences resulting in hyperglycemia, hyperosmolality, ketone production, and an acidic state.^[5]^ Several biochemical markers used to diagnose DKA include blood glucose (BG) levels > 11 mmol/L (or 200 mg/dL) and a venous pH of < 7.3 and/or actual bicarbonate (HCO_3_) level of < 15 mmol/L, ketonemia and ketonuria. DKA is resolved when the BG level is < 200 mg/dL, the pH is > 7.3, and the HCO_3_ level is 15 mEq/L or higher.^[5]^ High anion gap (AG) metabolic acidosis is a key symptom of DKA. This occurs because the body produces and builds up excessive amounts of ketoacids, such as hydroxybutyrate and acetoacetate.^[6]^ These ketones displace HCO_3_ in the blood, leading to a decrease in its concentration.^[6]^ Management strategies typically involve addressing the underlying cause of DKA, correcting fluid and electrolyte imbalances, and managing insulin therapy to mitigate the acidotic state and enhance patient outcomes.^[7,8]^ Electrolyte replenishment is largely performed with intravenous administration of normal saline (NS)^.[9]^ In addition, NS is the most commonly used fluid for insulin infusion preparation in most DKA management protocols.^[9]^ Due to its high chloride content (154 mmol/L) compared with human plasma (95–108 mmol/L), it can lead to hyperchloremic metabolic acidosis (HMA), especially when administered in large volumes.^[10]^

HMA is attributed to both the urinary excretion of ketones, which act as HCO_3_ precursors, with chloride retention, and the use of NS as a treatment, which further exacerbates the condition and hinders the restoration of acid–base balance.^[9]^ Consequently, careful monitoring and appropriate interventions are essential to restoring physiological conditions.

To our knowledge, no study has been published with the objective of evaluating the effect of changing insulin concentrations on the incidence of HMA and the time required for DKA resolution in pediatric patients. Therefore, this study aimed to primarily compare the effect of 2 insulin concentrations, 1 unit regular human insulin per 1 mL of 0.9% sodium chloride versus 1 unit regular human insulin per 10 mL of 0.9% sodium chloride, as part of DKA treatment, on the incidence of HMA. Secondary objectives include comparing the rate of cerebral edema (CE), total length of hospital stay, total chloride load (mmol/kg) in the first 24 hours, and time required for DKA resolution between the 2 insulin concentrations.

## 2. Methods

### 2.1. Study design

This retrospective observational study involved pediatric patients admitted to the pediatric intensive care unit or high-dependency unit at King Abdullah Specialized Children’s Hospital, a 600-bed tertiary hospital known as the Kingdom’s foremost and most advanced pediatric hospital, from January 2018 to June 2023. The inclusion criteria were pediatrics between the ages of 0 and 14 who were diagnosed with DKA and started on insulin infusions as per the DKA protocol. The exclusion criteria were patients who received DKA treatment that did not follow the institution protocol, patients in shock requiring vasopressors, and patients on mechanical ventilation. The sample size for this retrospective study was determined by including all available cases within the study period. In our institution, insulin for DKA management is administered at a dose of 0.1 units/kg/h as per the hospital protocol. In August 2020, an amendment was made to the insulin infusion preparation. Before this date, the insulin concentration was prepared by diluting 1 unit of human soluble insulin in 10 mL of 0.9% NS. However, after August 2020, the protocol was adjusted to increase the insulin concentration 10-fold, with a new preparation ratio of 1 unit of human soluble insulin per 1 mL of 0.9% NS. This modification aimed to minimize the total amount of chloride in the insulin preparation.

### 2.2. Data collection

Data included demographics, type of diabetes mellitus, severity of DKA, electrolyte levels, type of intravenous fluid used, total volume in the first 24 hours, total chloride load per kg in the first 24 hours, and length of hospital stay. The total chloride load (mmol/kg) is calculated by dividing the volume (in liters) and chloride concentration (in mmol/L) by the patient’s weight in kilograms. The severity of DKA is classified as follows: mild (pH 7.20–7.29 or HCO_3_ 10–15 mmol/L), moderate (pH 7.10–7.19 or HCO_3_ 5–10 mmol/L), and severe (pH < 7.10 or HCO_3_ < 5 mmol/L).^[11]^ DKA resolution is defined when BG levels fall below 200 mg/dL and at least 2 of the following conditions are met: a pH > 7.3, HCO_3_ levels exceeding 15 mmol/L, and an AG ≤ 12 mmol/L. HMA is defined as a condition with a pH < 7.35, a normal AG, and an increase in plasma chloride concentration > 108 mEq/L.

### 2.3. Ethical consideration

The study was approved by the ethics committee of the King Abdullah International Medical Research Center’s Institutional Review Board in April 2022 (NRC22R/172/04).

### 2.4. Statistical analysis

Data were summarized using descriptive statistics, including frequencies and percentages for categorical variables and means, standard deviation, median, and interquartile range for continuous variables. The normality of the data was assessed using the Shapiro–Wilk test. Comparison between the 2 groups for the pre- and postprotocol implementation was applied using the Student *t* test, Mann–Whitney *U* test, or *χ*^2^ test as appropriate. All statistical analyses were applied using the statistical software (SAS 9.4; SAS Institute Inc., Cary), and the significance level was declared at (*α* = .05).

## 3. Results

The study consisted of 279 admissions for DKA, with 140 admissions under the preprotocol and 139 under the postprotocol group. All patients were diagnosed with T1DM. There was a significant difference between the 2 groups in terms of the incidence of new T1DM cases, with 39% (54 patients) in the postprotocol group compared with 21% (30 patients) in the preprotocol group (*P* = .002). Regarding the severity of DKA, there were no differences in the distribution of mild, moderate, and severe DKA between the 2 groups. Moderate DKA was 43% (60 patients) in the preprotocol group and 37% (54 patients) in the postprotocol group. Severe DKA was 35% (49 patients) in the preprotocol group and 40% (56 patients) in the postprotocol group. Baseline biochemical data were similar between the 2 groups, except for initial sodium and chloride levels, which were higher in the postgroup (mean 132 meq/L ± 4 vs mean 133 meq/L ± 4; *P* = .001) (mean 101 meq/L ± 4 vs mean 103 meq/L ± 5; *P* = .001), respectively. In addition, the AG was higher in the pregroup (mean 28 ± 4 vs mean 26 ± 5; *P* = .002). Total fluid volume per kg received in the first 24 hours was similar between the 2 groups (mean 75 mL/kg ± 35 vs 74 mL/kg ± 31; *P* = .712). An overview of baseline demographics and biochemical data are provided in Table 1.

**Table 1:** Demographic and biochemical data.

Characteristics	Preprotocol (0.1 unit/mL) N = 140	Postprotocol (1 unit/mL) N = 139	*P* value
Age (year), median (IQR)	10 (7–15)	11 (9–11)	**.038**
Gender			.672
Female, N (%)	95 (68)	91 (65)	
Male, N (%)	45 (31)	48 (35)	
Weight (kg), median (IQR)	28 (22–38)	33 (25–45)	**.009**
Glasgow Coma Score	15 ± 1	15 ± 1	.483
New-onset diabetes			**.002**
New, N (%)	30 (21)	54 (39)	
Known, N (%)	110 (79)	85 (61)	
Severity of DKA			.596
Mild, N (%)	31 (22)	31 (22)	
Moderate, N (%)	60 (43)	52 (37)	
Severe, N (%)	49 (35)	56 (40)	
Type of IV fluid			.589
0.9% NaCl, N (%)	131 (94)	130 (94)	
0.9% NaCl and 0.45% NaCl, N (%)	8 (6)	9 (7)	
0.9 % NaCl and 3% NaCl, N (%)	1 (1)	0 (0)	
Initial bicarbonate (meq/L), mean ± SD*	7 ± 2	7 ± 2	.770
Initial glucose (mg/dL), mean ± SD*	28 ± 8	28 ± 8	.994
Initial sodium (meq/L), mean ± SD*	132 ± 4	133 ± 4	**.001**
Initial chloride (meq/L), mean ± SD*	101 ± 4	103 ± 5	**.001**
Initial anion gap, mean ± SD*	28 ± 4	26 ± 5	**.002**
Creatinine (µmol/L), mean ± SD	99 ± 24	96 ± 24	.398
Total fluid volume infused in the first 24 hrs (mL/ kg), mean ± SD	75 ± 35	74 ± 30	.712
Total fluid volume infused in the first 24 hrs (mL)	2062 ± 873	2336 ± 1046	**.014**
Insulin volume in the first 24 hrs (mL), mean ± SD*	363 ± 230	50 ± 32	**<.0001**

Bold value indicates statistical significance at *P* < .05.

DKA = diabetic ketoacidosis, IQR = interquartile range, IV = intravenous, SD = standard deviation.

* Level upon admission.

There was no difference in the incidence of HMA between the pre- and postprotocol groups. HMA was observed in 43% (60 patients) of the preprotocol group and 38% (53 patients) of the postprotocol group (*P* = .365).

**Figure 1.**
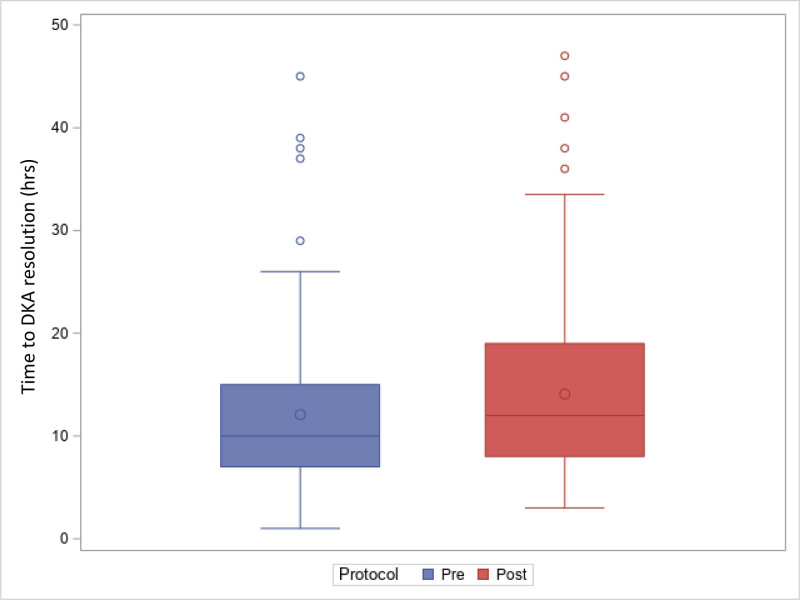
Time in hours to DKA resolution. DKA = diabetic ketoacidosis.

The total chloride load per kg in the first 24 hours did not differ significantly between the pre- and postgroups (mean 11 ± 5 and 11 ± 5, respectively; *P* = .665). The length of stay in days did not show a significant difference between the pre and postprotocol groups (mean 3 ± 2 vs 4 ± 3 respectively; *P* = .102) (Table 2). Patients in the postprotocol group, take longer to recover from DKA (*P* = .0053), as illustrated in Figure 1. Clinical CE defined by a low Glasgow Coma Score on presentation was identified only in 1 patient on the preprotocol group (Glasgow Coma Score = 4).

**Table 2:** Outcomes based on insulin concentration.

Characteristics	Preprotocol N = 140	Postprotocol N = 139	*P* value
Hyperchloremia, N (%)	60 (43%)	53 (38)	.365
CE, N (%)	1 (1%)	0 (0)	
Total chloride load: mmol/kg in the first 24 hrs	11 ± 5	11 ± 5	.665
pH, time to normalization	11 ± 6	13 ± 10	**.022**
HCO_3_, time to normalization	11 ± 7	14 ± 11	**.013**
AG, time to normalization	15 ± 11	13 ± 9	.128
length of stay (d), mean ± SD	3 ± 2	4 ± 3	.102

Bold value indicates statistical significance at *P* < .05.

AG = anion gap, CE = cerebral edema, HCO_3_, = actual bicarbonate.

## 4. Discussion

In this study, we address the important issue of reviewing DKA management protocols by examining the impact of changing insulin concentrations to minimize chloride content on the incidence of HMA and the time required for DKA resolution in pediatric patients. The rationale underlying our intervention included the use of more concentrated insulin to minimize the total amount of chloride and fluid in the insulin preparation to prevent hyperchloremia. However, in our study, although the posttest group showed a slight reduction in HMA, it was not statistically significant. Moreover, patients in the postprotocol group, take longer to recover from DKA. An explanation could be the higher prevalence of new-onset diabetes in the postgroup. The influence of new-onset diabetes on HMC is supported by a study conducted by Mrozik et al^[9]^, in which they evaluated markers of resolution in patients with moderate to severe DKA. Resolution criteria included a pH > 7.3, HCO_3_ levels exceeding 15 mEq/L, and an AG falling below 16 mEq/L. Their findings indicated that patients with newly diagnosed diabetes were at a higher risk of developing prolonged HMA compared to those with known diabetes (34% vs 13%, *P* < .05). It is also important to note that AG values are influenced by albumin levels, which can fluctuate in patients with DKA. Since hypoalbuminemia lowers AG, its variability should be considered when interpreting AG normalization trends. This factor may contribute to differences in the observed resolution rates between patient groups.^[12]^

In addition, it is important to note that the total amount of fluid infused into both groups was similar. In the postprotocol group, we reduced fluid volume by using concentrated insulin to reduce the chloride load. However, part of the fluid requirement was still provided to patients as per the protocol. This means that even though we aimed to decrease chloride levels through concentrated insulin, there was still some variability in the overall fluid balance due to the additional fluid given according to the protocol. In addition, our analysis showed that the insulin-containing saline volume significantly decreased in the postprotocol group (from 363 ± 230 to 50 ± 32 mL; *P* < .0001). However, this represented only 2% of the total fluid volume in the postprotocol group compared with 18% in the preprotocol group.

Studies have evaluated different strategies to minimize or prevent HMA. In 1 retrospective chart review, including 123 pediatric patients admitted with DKA, who received either only NS or half NS, they found a significantly higher incidence of hyperchloremia leading to non-AG metabolic acidosis in the NS group compared with the half NS group (*P* < .01).^[13]^

Moreover, a recent systematic review and meta-analysis of 8 randomized controlled trials (RCTs) included patients with DKA resituated with either saline or balanced crystalloid, and they concluded that the administration of a high volume of NS may be associated with a higher rate of hyperchloremia and a longer time to DKA resolution.^[14]^ In addition, a recent RCT investigated the impact of different fluid rates and their effect on electrolyte levels in 714 children undergoing treatment for DKA. They found that chloride serum increased more rapidly with NS versus half saline, and also increased the incidence of hyperchloremic acidosis in patients in the fast administration rate group.^[15]^ Studies have shown that hyperchloremia is an independent risk factor for both 28-day mortality and persistent organ dysfunction in pediatric patients with septic shock.^[16,17]^ This highlights the importance of considering and managing chloride levels when administering intravenous fluids to pediatric patients. In a large RCT investigating fluid treatment for pediatric DKA, it was found that the arms receiving 0.45% NaCl exhibited a lesser increase in chloride levels compared with those receiving 0.90% NaCl, resulting in a decrease in the rate of hyperchloremic acidosis.^[15]^ Therefore, if there is a need to supplement fluid while reducing the chloride load, the use of 0.45% NaCl may be a favorable option. In our study, although the lack of significant differences in the incidence of hyperchloremic metabolic total chloride load in the first 24 hours and hospital stay between the 2 protocols, the use of concentrated, supported by existing literature,^[5,7]^ is more convenient for the nurses which can enhance efficiency in clinical settings. Clinicians, particularly pediatric specialists, are cautious about modifying the standard DKA NS fluid regimen due to concerns about causing hyponatremia and subsequent CE.^[9]^ Although CE in pediatric DKA is associated with a significant mortality rate,^[18,19]^ it is important to highlight that this rare case^[18,19]^ should not discourage clinicians from seeking alternative methods to improve DKA management protocols.

The study’s limitation lies in the retrospective nature of data collection. However, all patients received consistent management in accordance with local DKA guidelines, ensuring standardized treatment, monitoring, and criteria for evaluating DKA resolution. Further studies with larger sample sizes, considering other fluid options, are recommended to validate these findings. In our study, the consistency of laboratory methods across the study period is a notable strength, as there were no changes in how sodium, chloride, or AG values were measured. This consistency helps ensure the reliability and accuracy of our biochemical data. However, we acknowledge that advances in laboratory techniques over time can influence values such as chloride and AG, potentially affecting study results. Although not applicable in our case, this factor should be considered when comparing results with other studies where lab methods may have evolved. We recognize this as a limitation, as any undetected variability in lab protocols could contribute to type 2 errors. In addition, our study was conducted using traditional definitions of HMA and DKA resolution. It cannot be excluded that applying alternative perspectives, such as the Na–Cl gap or the Stewart approach, might have resulted in different interpretations of the findings.^[20]^ Finally, we acknowledge that this study was not registered before data collection.

## 5. Conclusion

Our study found no significant difference in the incidence of HMA between the 2 insulin concentrations used in the treatment of DKA. This suggests that changing insulin concentrations does not impact the occurrence of this condition. Physicians should carefully weigh the benefits and risks of each insulin concentration when managing DKA.

## Acknowledgments

We thank Princess Nourah Bint Abdulrahman University Researchers Supporting Project number (PNURSP2025R484), Princess Nourah Bint Abdulrahman University, Riyadh, Saudi Arabia.

## Author contributions

**Conceptualization:** Hessa Al Muqati, Abeer Alsmari, Mohanned Alrahili, Fahad Al Juraibah, Omar AlDibasi, Nada A. Alsaleh.

**Data curation:** Hessa Al Muqati, Abeer Alsmari, Mohanned Alrahili, Fahad Al Juraibah, Omar AlDibasi, Nada A. Alsaleh.

**Formal analysis:** Hessa Al Muqati, Abeer Alsmari, Mohanned Alrahili, Fahad Al Juraibah, Omar AlDibasi, Nada A. Alsaleh.

**Investigation:** Hessa Al Muqati, Abeer Alsmari, Mohanned Alrahili, Fahad Al Juraibah, Nada A. Alsaleh.

**Methodology:** Hessa Al Muqati, Abeer Alsmari, Mohanned Alrahili, Fahad Al Juraibah, Omar AlDibasi, Nada A. Alsaleh.

**Resources:** Hessa Al Muqati, Abeer Alsmari, Mohanned Alrahili, Fahad Al Juraibah, Omar AlDibasi.

**Software:** Hessa Al Muqati, Abeer Alsmari, Omar AlDibasi, Nada A. Alsaleh.

**Visualization:** Hessa Al Muqati, Abeer Alsmari, Mohanned Alrahili, Fahad Al Juraibah, Omar AlDibasi.

**Writing – original draft:** Hessa Al Muqati, Abeer Alsmari, Mohanned Alrahili, Fahad Al Juraibah, Omar AlDibasi, Nada A. Alsaleh.

**Writing – review & editing:** Hessa Al Muqati, Abeer Alsmari, Mohanned Alrahili, Fahad Al Juraibah, Omar AlDibasi, Nada A. Alsaleh.

**Project administration:** Abeer Alsmari.

**Supervision:** Abeer Alsmari, Mohanned Alrahili, Fahad Al Juraibah, Nada A. Alsaleh.

**Validation:** Abeer Alsmari, Mohanned Alrahili, Fahad Al Juraibah, Omar AlDibasi, Nada A. Alsaleh.

**Funding acquisition:** Nada A. Alsaleh.
